# Maintenance of Functional CD57+ Cytolytic CD4+ T Cells in HIV+ Elite Controllers

**DOI:** 10.3389/fimmu.2019.01844

**Published:** 2019-08-08

**Authors:** Chansavath Phetsouphanh, Daniel Aldridge, Emanuele Marchi, C. Mee Ling Munier, Jodi Meyerowitz, Lyle Murray, Cloete Van Vuuren, Dominique Goedhals, Sarah Fidler, Anthony Kelleher, Paul Klenerman, John Frater

**Affiliations:** ^1^Peter Medawar Building for Pathogen Research, University of Oxford, Oxford, United Kingdom; ^2^Department of Medicine, University of New South Wales, Sydney, NSW, Australia; ^3^Military Hospital, Bloemfontein, South Africa; ^4^National Health Laboratory Service, Division of Virology, University of the Free State, Bloemfontein, South Africa; ^5^Imperial College London, London, United Kingdom

**Keywords:** T cells, HIV, viral immunology, CD4 T cells, cytotoxic T cells

## Abstract

Cytolytic CD4+ T cells play a prominent role in chronic viral infection. CD4+ CTLs clones specific for HIV-1 Nef and Gag are capable of killing HIV-1 infected CD4+ T cells and macrophages. Additionally, HIV-specific cytolytic CD4+ T cell responses in acute HIV infection are predictive of disease progression. CD57 expression on CD4s identifies cytolytic cells. These cells were dramatically increased in chronic HIV infection. CD57 expression correlated with cytolytic granules, granzyme B and perforin expression. They express lower CCR5 compared to CD57– cells, have less HIV total DNA, and were a minor component of the HIV reservoir. A small percentage of CD57+ CD4+ CTLs from EC were HIV-specific, could upregulate IFNγ with Gag peptide stimulation, express cytolytic granule markers and maintain Tbet^high^Eomes+ transcription factor phenotype. This was not observed in viraemic controllers. The maintenance of HIV-specific CD4 cytolytic function in Elite controllers together with CD8 CTLs may be important for the control of HIV viraemia and of potential relevance to cure strategies.

## Introduction

Observations have been made since the late 1970s that CD4+ T cells are not merely helper cells, but can also have cytolytic activity (1, 2). Allogeneic responses originally described in murine models highlighted these observations, and were further shown in human memory CD4+ T cell recall responses to persistent viral infections. Cytolytic CD8+ T cells and neutralizing antibodies produced by B cells usually control chronic pathogens that infect immune cells, but some pathogens (i.e., CMV, EBV, and HIV) have evolved mechanisms to avoid this control, primarily by the down-regulation of HLA Class I molecules (3). However, the immune targets of these pathogens constitutively express HLA Class II that represents a potentially direct focus of CD4 effector mediated control (4).

Containment of viral replication by cytolytic CD4+ T cells (CD4 CTLs) has often been overshadowed by the presence of CD8 CTLs that outnumber them. CD8 CTLs have long been perceived as the main contributor of control in acute and chronic viral infections, however their role is limited by viral escape mechanisms. Many such escape mechanisms linked to incomplete CTL-mediated control have been described, which suggests that there may be an alternate cytolytic pathway contributing to viral control (5). CD4 CTLs that recognize cognate viral epitopes through MHC class II engagement may act in concert with CD8 CTLs to further strengthen viral control (6). Studies on Dengue virus (7), HPV and HIV-1 (8) have shown that CD4 CTLs cells are activated and highly cytolytic and may contribute to prevent disease progression. CD4 CTLs has also been shown to play a pivotal role in the containment of viral replication in Influenza, EBV and CMV infections (9).

CD4 CTLs were previously observed in HIV infection by Appay et al. and were defined as antigen experienced, terminally differentiated with CD8 CTLs-like phenotype i.e., CD27–CD28–CD45RO+CCR7-perf+gzmA+, and that their killing mechanism was granzyme and perforin dependent (10, 11). This was confirmed by a study that observed in acute HIV infection that CD57+ CD4+ T cells were predominantly perforin+, granzyme B+ and Eomes+ (8), which suggests that CD57 expression may identify cytolytic CD4+ T cells. CD4+ CTLs clones specific for HIV Gag were generated from seronegative donors; these clones together with Nef-specific CD4+ CTLs clones were shown to be capable of killing HIV-1 infected CD4+ T cells and macrophages (12). The presence of these HIV-specific cytolytic CD4+ T cell responses in acute HIV infection was highly predictive for disease outcome (13). Maintenance of functional HIV-specific CD4 CTLs in Long-Term Non-Progressors (LTNP) may add another level of immune protection against the virus.

CD4 CTLs cells are long-lived and have been shown in many viral infections to be effective in the elimination of infected target cells. However, little work has been undertaken to understand their role in HIV infection. We hypothesize that there is loss of CD4 CTLs function during HIV infection. CD57 was utilized as a marker of CD4 CTLs to investigate their phenotype and function during different stages of HIV infection, that is, from primary to chronic infection. CD57 expressing CD4+ T cells have a transcription signature that closely resembles CD8 CTLs. Functional CD4 CTLs were maintained in HIV Elite Controllers, and not in Viraemic Controllers. CD4 CTLs may potentially act in concert with CD8 CTLs to control HIV viraemia, and may be an important factor that distinguishes Elite and Viraemic control.

## Methods

### Participant Samples

Participants with Primary HIV (PHI) were recruited as either part of the HEATHER (HIV Reservoir targeting with Early Antiretroviral Therapy) cohort or from the SPARTAC (Short Pulse Antiretroviral Therapy at HIV Seroconversion) trial (EudraCT Number: 2004-000446-20). For inclusion in the HEATHER cohort, participants with identified PHI commenced ART within 3 months of diagnosis, and did not have co-infection with Hepatitis B or C. For our study, cryopreserved PBMCs were used from the closest pre-therapy sample to seroconversion (baseline) and from a sample 9–15 months after commencement of ART (1 year). Only Baseline samples were used from the SPARTAC trial, which was a multi-center, randomized controlled trial of short course ART during PHI, the full design of which is described elsewhere (38).

Participants with Chronic HIV (CHI) were recruited in Bloemfontein, located within the Mangaung Metropolitan Municipality in the Free State province of South Africa. Most participants had advanced HIV-1 disease progression (as reflected by a CD4 T cell count <350 cells/μL). All participants were tested for HIV-1 using a point-of- care “HIV-1 rapid test” or laboratory-based HIV-1 ELISA. Follow up samples were collected at 6 and 12 months post-ART initiation (39).

LTNPs samples were collected at various sites across New South Wales, Australia; samples were processed and stored at St. Vincent's Centre of Applied Medical Research, Darlinghurst. Eligible subjects were HIV+, asymptomatic and diagnosed at least 8 years previous to enrolment, treatment naïve, and had an absolute CD4+ T cell count ≥ 500 cells/μL. LTNPs were divided in to two groups: Elite Controllers (EC) with undetectable viral load (median of 1.7 Log) whilst LTNPs with detectable viral load (median of 5.18 Log) were designated Viraemic controllers (VC) and used as comparators to EC (Table 1). The St. Vincent's Research Ethics Committee (EC00140) approval number: HREC/12/SVH/298, SVH 12/217. PBMCs obtained from healthy donors were approved by the Sheffield Research Ethics Committee (reference 16/YH/0247). All participants from each of the above mentioned cohorts gave informed consent for their participation in these studies.

**Table 1:** Sample cohort characteristics.

**Patient cohort**	**Sample number**	**CD4+ T cells (count/μL) Median (IQR)**	**Plasma viral load (Log_**10**_ copies/mL) Median (IQR)**
Healthy donors	19	–	–
PHI (SPARTAC)	22	596 (437–755)	5.04 (4.51–5.45)
PHI (HEATHER)	12	524 (437–656)	4.34 (3.19–4.88)
CHI	13	379 (308–763)	4.89 (4.10–5.59)
EC LTNP	9	780 (615–1,013)	1.70 (1.60–1.70)
VC LTNP	10	633 (442–800)	5.18 (5.03–5.37)

*Median CD4+ T cells counts/μL with interquartile ranges. Median plasma viral load (Log_10_ copies/mL) with interquartile ranges*.

### Flow Cytometry

Frozen PBMCs were thawed using R10 medium (RPMI+L-glutamine+Penicillin Streptomycin+ 10% FCS) and subsequently stained with antibodies corresponding to either the chemokine/cytokine receptor, cytolytic, or transcription factor panels (see below). FoxP3 permeabilization kit (BD Pharmingen) was used for intracellular staining. Staining of the chemokine panel was carried out at 37°C. Samples were acquired on an LSRII flow cytometer (BD Biosciences) using the FACSDiva software package (BD Biosciences). Prior to each run, all samples were fixed in 2% PFA. Samples were then analyzed using the Flowjo software package (FlowJo, LLC). Gating strategies were developed based on florescence-minus-one (FMO) controls.

#### Base Panel

Live/Dead dye (Invitrogen), CD4 (RPA-T4, BD Biosciences), CD3 (UCHT1), CD8 (5K1) and CD57 (HNK-1)[all Biolegend]. Added to all flow cytometry-staining panels.

#### Chemokine/Cytokine Receptor Panel

CCR5 (2D7/CCR5), CXCR3 (1C6/CXCR3,), CD25 (M-A251), CCR6 (11A9)[all BD Biosciences), IL-15R (eBioJM7A4, eBiosciences), CD127 (A019D5), CXCR4 (L276F12), CCR4 (L291H4)[all BD Biosciences]. Chemokine receptors stained better at 37°C for 15 mins.

#### Cytolytic Panel

Granzyme B (GB11), CD107a (H4A3)[BD Biosciences], Granzyme A (CB9), Perforin (B-D48)[Biolegend].

#### Transcription Factor Panel

Tbet (eBio4B10), Eomes (WD1928)[eBiosciences].

### Intracellular Cytokine Staining Assay

Frozen PBMCs were thawed using IMDM+10% heat inactivated AB serum medium. PMBCs were cultured in 24-well plates and rested for 3 h at 37°C. Individual cultures were stimulated with CMV pp65 peptides at a final concentration of 10 μg/ml; or HIV Clade B gag pool of 123 15mer overlapping peptides (NIH AIDS Research and Reference Reagent Program) used at a final concentration of 10 μg/ml for each peptide. 10 μg/ml of Brefeldin A (Sigma-Aldrich) was added after 2 h of stimulation with antigen. Cultures were incubated at 37°C overnight in a humidified atmosphere with 5% CO_2_ in air. Negative control cultures comprised PBMCs mixed with IMDM with 10% heat inactivated AB serum only, while SEB (5 μg/mL) was used for positive control cultures (40). Intracellular staining included addition of the base panel prior to permeabilization, followed by cytolytic panel, as well as IFNγ (B27, Biolegend).

### Cytokine Bead Array Assay

CD57+ and CD57– CD4 T cells were sorted using the MoFlo XDP cell sorter (Beckman Coulter). Cells were isolated based on their positive expression of CD3, CD4, CD45RO, and CD57. Sorted cells had >98% purity (Supplementary Figure 1). Sorted cells were then incubated overnight with or without PMA (50 ng/mL) and Ionomycin (1 μg/mL) at 37°C. Supernatant were harvested from the cultures and used for the Cytokine Bead array Assay (CBA). IFNγ, TNF, IL-10, FASL, GzmB, and MIP-1β analytes were examined according to the manufacturers instructions on the BD LSRII (Becton Dickinson).

### RNA Isolation and qPCR

CD45RO+CD57+ and CD45RO+CD57– cells were sorted from 3 separate donors. RNA was then isolated from these cells using a TRIzol reagent based technique. TRIzol (Life Technologies) was added to cells to lyse them and dissolve the cell contents. Chloroform was added and the solution was centrifuged at 12,000 × g to separate the dissolved cellular contents into differing layers. The aqueous layer containing RNA was then extracted, incubated with glycogen capture protein and isopropanol before being washed with isopropanol again. After washing, the isopropanol was driven off by evaporation and RNA was resuspended in DNAse/RNAse free water. RNA concentration and purity was then determined using a Bioanalyzer RNA kit (Aligent Genomics) and run on an Aligent Bioanalyzer 2100 instrument. DNAse treatment step was performed prior to RNA-seq (Thermo-fisher). Real-time PCR (RT-PCR) was performed using the Light Cycler 480 system (ROCHE). Taqman primers/probes used include ACTB (Hs001060665_g1), GzmB (Hs00188051_m1) and CCR5 (Hs00152917_m1).

### RNA-Seq

RNAseq analysis was performed by the Wellcome Trust Centre for Human Genetics. All samples were normalized to 110 ng, as 100 ng was the minimum amount that was needed for the procedure and 110 ng was the value of our samples closest to this. The Wellcome Trust Centre (Oxford, UK) then performed library preparation using poly-A selection to isolate mRNA transcripts, which were then converted to cDNA and sequenced by the Illumina HiSeq4000 platform with 75bp ended reads. The raw sequencing data were mapped to a human genome reference (hg19) using the STAR alignment software. Read counts matrix, data preprocessing, and statistical analysis was done in R environment language employing Bioconductor packages.

### DNA Isolation and Total-HIV DNA PCR

For measurement of HIV DNA in bulk CD4 T cells, CD4 T cells were enriched from cryopreserved PBMCs as above or using Dynabeads Untouched Human CD4 T Cell Enrichment kit (Invitrogen). DNA was extracted from PBMCs or enriched CD4 T cells using QIAamp Blood Mini Kit (Qiagen) and sorted CD4 T cell subsets (QIAmp DNA Micro Kit or Mini Kit) for use as input for qPCR assays. Copies of HIV-1 DNA were quantified and normalized to number of input cells (as determined by albumin PCR), by a previously described assay (41, 42) with both qPCR assays performed in triplicate. For sorted populations PCR reactions for both albumin and total HIV were performed in triplicate for the CD32- population and in duplicate for the remaining populations, except where otherwise noted. Negative sample wells were replaced with zeros when averaging replicate values.

### Cellular Proliferation Assay

Sorted cells were stained with Cell Trace Violet dye (Invitrogen), according to the manufacturer's protocol, and were subsequently stimulated with antigens, 5 μg/mL SEB (Sigma), 10 μg/mL CMVpp65 peptide and 10 μg/mL HIV gag peptide pool (NIH, USA). Cells were incubated for 6 days and stained with florescent antibodies and analyzed by flow cytometry. 100IU of IL-2 (Invitrogen), 20 ng/mL of IL-12 and 20 ng/mL of IL-18 (Peprotech) were used to stimulated CD4+CD57+ cells together with 5 ug/mL of anti-CD3 (BD biosciences).

### Cytoxicity Assay

CD57+CD4+ (Effector) T cells were sorted together with autologous CD19+ (targets) B cells using Beckman Coulter MoFlo. Target cells were stained with Cell Trace Violet and rested together with effector cells overnight in media at 37°C. Differing effector to target cell ratios were used (i.e., 1:1, 2:1, and 3:1), a minimum 10,000 targets cells were used for each condition, with targets only as a negative control. Live/dead dye was used to stain dead cells from overnight culture before 5 μg/mL of anti-CD3 beads (Miltenyi) was added to the cultures for 3.5hrs. Killing percentage was calculated by measuring the expression of caspase 3 (CP92-605, BD) within target cells minus negative control.

### Statistical Analysis

All column graphs are presented as medians with inter-quartile ranges. Wilcoxon paired *t-*test was used to analyze statistical data employing the Prism 7.0 (GraphicPad, La Jolla, CA, USA) software. For unpaired samples the Mann-Whitney *U* test was used. *p* < 0.05 were considered significant.

## Results

### CD57 Expression Identifies Cytolytic CD4+ T Cells

**Figure 1:**
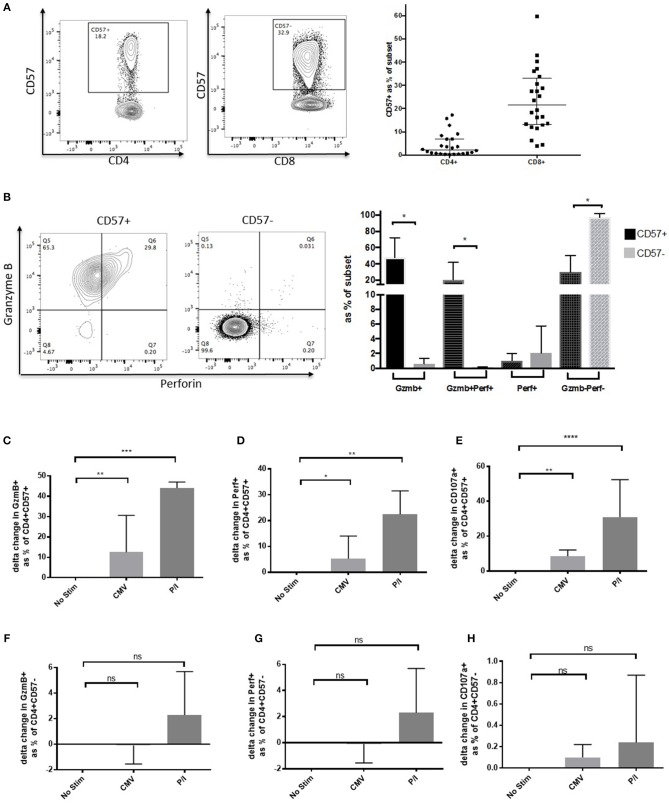
CD57 identifies Cytolytic CD4+ T cells. **(A)** Representative dot plot showing CD57 expression on CD4+ and CD8+ T cells. Distribution of CD57 levels in healthy donors (*n* = 24). **(B)** Dot plot and column graph showing Granzyme B and Perforin expression in CD57+ and CD57– CD4+ subsets in healthy donors. **(C)** Upregulation of Granzyme **(B,D)** Perforin and **(E)** CD107a after 6 h of activation with CMV and PMA/ionomycin in the CD4+CD57+ subset (*n* = 6). **(F–H)** Expression of cytolytic markers in CD4+CD57– subset 6 h post activation (**F** = Granzyme B, **G** = Perforin, and **H** = CD107a). *p < 0.05, **p < 0.01, ***p < 0.001, ****p < 0.0001.

To address whether CD57 could be used as a marker for CD4 CTLs, we stained for its surface expression on PBMCs from 24 healthy donors. There was a median of 3.56% (IQR: 0.66–17.21%) within the CD4+ subset and ten-fold higher expression in the CD8+ subset [21.5% (13.03–59.64%)] (Figure 1A). To show that these CD57+ CD4+ T cells were potentially cytolytic, we next stained for cytolytic granule markers Granzyme B and Perforin. The majority of GzmB+ (48.01%, *p* < 0.05) expression was found in the CD57+ subset, as well as a considerable percentage of activated GzmB+Perf+ (20.74%, *p* < 0.05), both were significantly higher than that observed in the CD57– counterpart (Figure 1B). The CD57– subset was overwhelmingly GzmB-Perf- (92.70%), which suggests that CD57 expression identifies the majority of cytolytic CD4+ T cells. Gzm A was co-expressed with GzmB in CD4+CD57+ cells and they could also express different combinations of these cytolytic granules depending on activation state (Supplementary Figure 2A).

To elucidate whether CD4+CD57+ could further upregulate cytolytic granule expression when activated, PBMC from healthy donors were stimulated with either CMVpp65 or PMA/Ionomycin (P/I) for 6 h. Delta change from no stimulation was used to determine increase of GzmB expression following activation. Median increase of GzmB from no stim was 12.5% for CMVpp65 and 44.1% for P/I (*p* < 0.01 and *p* < 0.001, respectively; Figure 1C). An increase was also observed with perforin expression (5.2%, *p* < 0.05 [CMV] and 22.4%, *p* < 0.01 [P/I]; Figure 1D). To assess potential for degranulation we measured CD107a expression post-activation showing an increase on stimulation (8.5%, *p* < 0.01 [CMV] and 30.9%, *p* < 0.001 [P/I]; Figure 1E). These significant increases were not observed within the CD57– subset (Figures 1F–H). CD57+ CD4 CTLs have a unique phenotype, they do not express the typical Treg phenotype CD25^high^CD127^low^ and have low expression of CD127, CCR4, CCR6 and CXCR3 *ex vivo* [CD4 lineage phenotype: Th17 (CCR4+ and CCR6+) and Th1 (CXCR3+ CCR6–) subsets] (Supplementary Figure 2B).

### CD57+ CD4 CTLs Can Proliferate and Are Antigen-Specific

**Figure 2:**
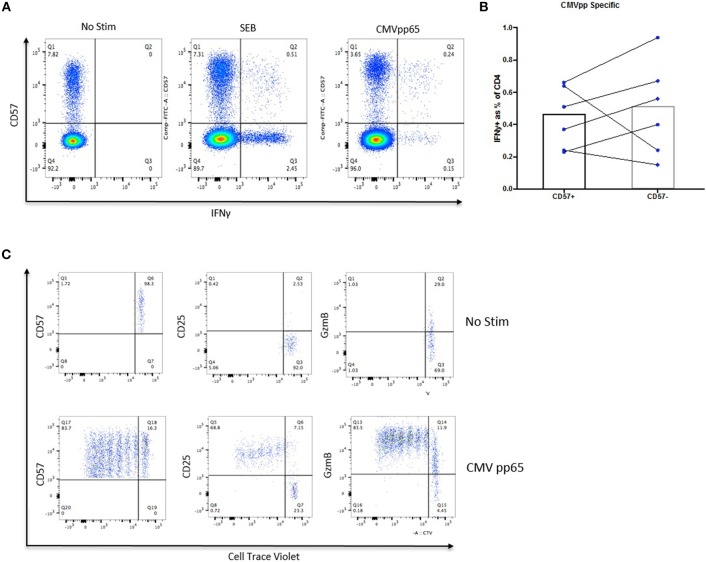
CD57+ CD4 CTLs can proliferate and are antigen-specific. **(A)** IFNγ expression in SEB- and CMV-specific CD57+ CTLs. **(B)** No difference between IFNγ expression between CMV-specific CD57+ and CD57– susbets. **(C)** Proliferation of CMV-specific CD57+ from healthy donors.

The ICS assay was utilized to determine whether CD4 CTLs were antigen-experienced and had the ability to respond to recall antigens when stimulated. CD4+CD57+ cells produced IFNγ in response to super-antigen SEB and CMVpp65, although with comparable levels to those observed in the CD4+CD57– subset (0.45% and 0.5%, respectively; Figures 2A,B). CD57+ Cells were also able to degranulate and up-regulate CD107a, while maintaining GzmB and IFNγ expression when stimulated with CMVpp65 and SEB. IFNγ+ CD57– cells did not up up-regulate CD107a or Granzyme B (Supplementary Figure 3A). CD4+ CD57+ cells were sorted and stimulated with CMVpp65 for 6 days to determine the proliferation potential of these cells. CD57 and GzmB expression was maintained in daughter cells, while up-regulation of CD25 (IL-2Rα) was observed following cell division (Figure 2C). This suggests that IL-2 is required for CD4+CD57+ proliferation. IL-2 together with IL-12 and IL-18 were added *in vitro* to drive CD4+CD57+ proliferation. The combination of IL-2 with IL-12 was sufficient for the proliferation of CD4+CD57 cells (Supplementary Figure 3B). Immobilized anti-CD3 stimulation alone was not sufficient in driving proliferation, but this was rectified with the addition of IL-2 and IL-12 (Supplementary Figure 3C). CD4+CD57– cells were sorted into naïve and memory subsets based on CD45RO expression. Both subsets proliferated with anti-CD3/CD28 stimulation but did not up-regulate CD57 expression (Supplementary Figure 3D).

### CD57+ CD4+ T Cells Have a Cytolytic Transcription Profile

**Figure 3:**
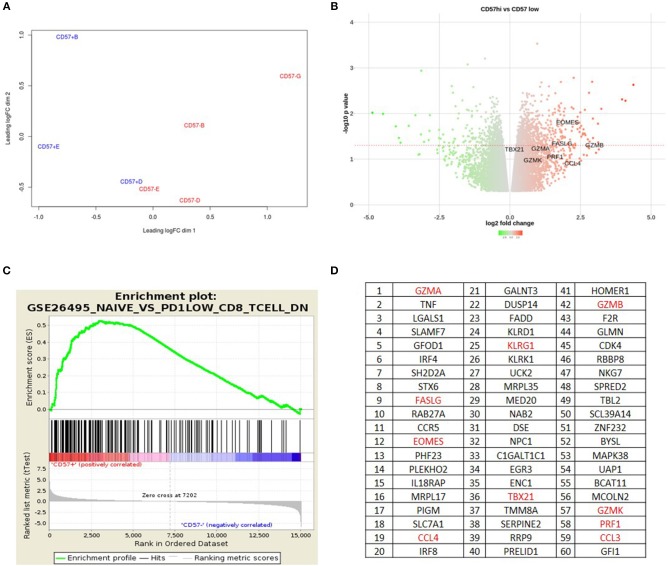
CD57+ CD4+ T cells have a cytolytic transcription profile. **(A)** Principle Component Analysis. **(B)** Volcano plot showing over and under-expressed genes in CD4+CD57+RO+ vs. CD4+CD57–RO+. **(C)** GSEA plot showing CD4+CD57+RO+ correlating with PD-1^low^CD8+ T cells. **(D)** Leading Edge Analysis showing top 60 genes expressed in CD4+CD57+RO+ subset.

To examine the transcriptome of CD57+ CD4+ T cells, mRNA was extracted from four healthy donors and RNAseq was performed. CD45RO+CD57– paired samples were used as a comparator group. CD57+ and CD57– transcription profiles clustered separately through Principal Component Analysis (PCA) (Figure 3A). Over-expressed genes with the CD57+ subset included cytolytic granule genes and transcription factors Tbet and Eomes (Figure 3B). Differentially expressed genes from our CD57+ dataset positively correlated with CD8+ datasets using Gene Set Enrichment Analysis (GSEA) (Figure 3C). Leading-edge analyses was performed to determine the most differentially expressed genes in our dataset compared to other publicly available datasets with a normalized false discovery rate of <0.03. The top 60 expressed genes in our CD57+ subset were predominantly cytolytic associated genes including *gzma, fslg, eomes, ccl4, tbx21, gzmb, gzmk and prf1* (Figure 3D).

To confirm the GSEA data that CD57+ CD4+ correlated highly with a CD8+ transcription profile, RUNX3 and ThPOK expression was examined. RUNX3 is an important transcription factor required for CD8 lineage commitment (14). RUNX3 mRNA levels were much higher in CD57+ CD4+ subsets compared with CD57–, comparable to CD8+ subsets (Supplementary Figure 4A). This was also observed at the protein level, where CD57+ cells had higher MFI (Supplementary Figure 4B). The CD4 lineage transcription factor ThPOK (15) mRNA expression was lower in CD57+ CD4+ subsets compared with CD57– (Supplementary Figure 4C). Collectively these data demonstrate that CD57+ CD4+ cells have a cytolytic transcription profile similar to cytolytic CD8+ T cells.

### Accumulation of CD57+ CD4+ T Cells in Chronic HIV Infection

**Figure 4:**
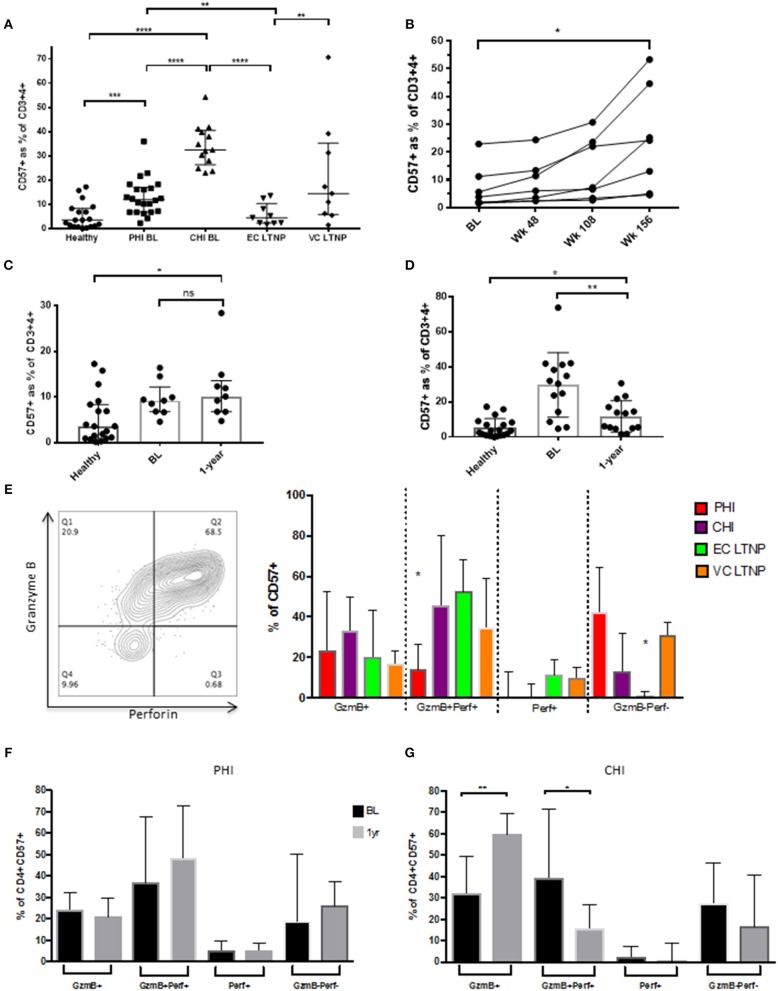
Accumulation of CD57+ CD4 CTLs in HIV infection. **(A)** CD57 distribution between HIV infected cohorts. **(B)** Increased CD57 subset from baseline to week 156 in primary HIV (SPARTAC) standard of care arm (*n* = 7). **(C)** No difference between CD57 frequencies at baseline and 1-year post-ART in PHI. **(D)** Decreased CD57 levels 1-year post-ART in CHI, with healthy donor as reference. **(E)** Granzyme B and Perforin expression in CD57 subset in differing stages of HIV infection. **(F)** No difference in GzmB and Perf expression PHI 1-year post-ART. **(G)** Increase of GzmB and decrease GzmB+Perf+ post-ART in CHI. *p < 0.05, **p < 0.01, ***p < 0.001, ****p < 0.0001.

Next we sought to investigate CD57+ CD4+ CTLs at different stages of HIV infection, as well as during Long-Term Non-Progression. CD57+ CD4 CTLs frequencies were much higher during untreated acute/primary HIV than in HIV-uninfected (3.42%) individuals and even higher still in untreated chronic infection (median of 12 and 32.5%, *p* < 0.0001, respectively). Differences were observed in the LTNP cohort, whereby Elite Controllers (EC) had low percentages (4.52%) of these cells similar to healthy donors (3.42%), whereas viraemic controllers (VC) were considerably higher (14.5%; *p* < 0.01; Figure 4A). These data suggest that an accumulation of CD4 CTLs occurs in chronic HIV infection. In order to elucidate this observation in further detail, we used paired samples from the standard-of-care arm of the SPARTAC trial, 12 participants were followed from early in infection (acute phase), who received no therapy and were followed up longitudinally for 156 weeks (chronic phase). As previously observed, there was a gradual increase of the CD57+ subset within the total CD4+ T cells from baseline to week 156 (3.85 to 24.2%; *p* < 0.05; Figure 4B). No difference was observed after 1 year of ART and basline PHI (Figure 4C), but a significant drop was seen following 1-year of ART during CHI (~3.2-fold; *p* < 0.01), CD57 expression remained significantly higher than healthy donors (*p* < 0.05; Figure 4D). CD57+ cells were also highly activated with increased expression of GzmB+Perf+ during chronic infection compared to PHI (~2.3-fold, *p* < 0.05; Figure 4E). There was no difference between baseline and 1year post-Tx for PHI (Figure 4F), but a significant increase in Gzmb+ (~1.9-fold; *p* < 0.01) was observed in CHI post therapy together with a decrease in GzmB+Perf+ (~2.5-fold, *p* < 0.05; Figure 4G).

### CD57+ CD4 CTLs in HIV Infection Are Effector Memory Cells With a Tbet^high^Eomes+ Phenotype

**Figure 5:**
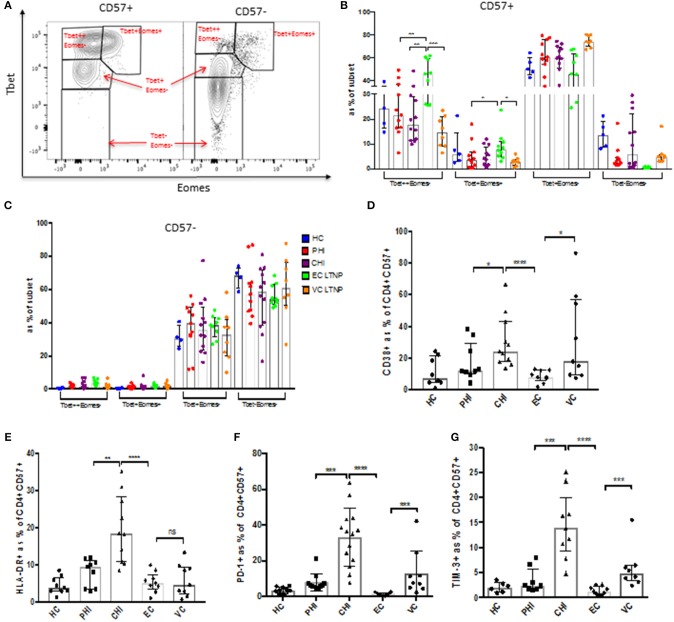
CD57+ CD4 CTLs have a Tbet^high^Eomes+ phenotype. **(A)** Representative dot plot of Tbet vs. Eomes expression. **(B,C)** Tbet and Eomes expression in CD57+ and CD57– in CD4+ cells during different stages of HIV infection. **(D)** CD38 expression. **(E)** HLA-DR protein levels. **(F)** PD-1 inhibitory receptor expression. **(G)** TIM-3 expression. *p < 0.05, **p < 0.01, ***p < 0.001, ****p < 0.0001.

Expression of CD57 on T cells is generally associated with terminal differentiation and the reduction of co-stimulatory receptor expression. CD28 is required for full T cell activation and is an important co-stimulatory molecule. During HIV infection CD28 expression decreases on CD57+ cells (Supplementary Figure 5A), which suggests that they may utilize other co-stimulatory molecules for activation. Central (Tcm) to effector memory (Tem) transition was observed from PHI to CHI, demonstrating a reduction in the proportion of CD4+ T cells expressing CD45RA and CCR7 following HIV infection (Supplementary Figures 5B,C). Transcription factors Tbet and Eomesodermin (Eomes) are important for cytolytic function (16, 17). CD57+ CD4 T cells have a distinct profile compared to their CD57– counterparts (Figure 5A). They were primarily Tbet+, Tbet++(high) and Tbet+Eomes+, whereas CD57– cells were mostly Tbet negative (potentially encompassing other T helper subsets) and have very little Tbet++ or Eomes expression (Figures 5B,C).

Activation markers and immune checkpoint receptors (ICRs) were also markedly increased on CD57+ cells in chronic infection (Supplementary Figure 6A). CD38 and HLA-DR activation markers were highly expressed in CHI (baseline) compared to the other cohorts, with ECs having very low activation markers comparable to healthy controls (Figures 5D,E). CHI and VC had higher expression of ICRs PD-1 and Tim-3 (Figures 5F,G). There was a slight decrease of CD38 (~1.5-fold, *p* < 0.05) 1 year post-ART in PHI subjects, but no change was observed in HLA-DR or PD-1 expression (Supplementary Figure 6B). Tbet and Eomes expression was not altered following ART in primary infection (Supplementary Figure 6C). CD57+ CD4 CTLs express high levels of fractalkine receptor CX3CR1, as well as the inhibitory receptor 2B4 (CD244). Although they do not co-express previously identified cytolytic markers CrTAM and SLAMF7 (Supplementary Figures 6D,E).

### CD57+ CD4 CTLs Express Low Levels of CCR5 and CXCR4, and Are Not a Major Component of the HIV Reservoir

**Figure 6:**
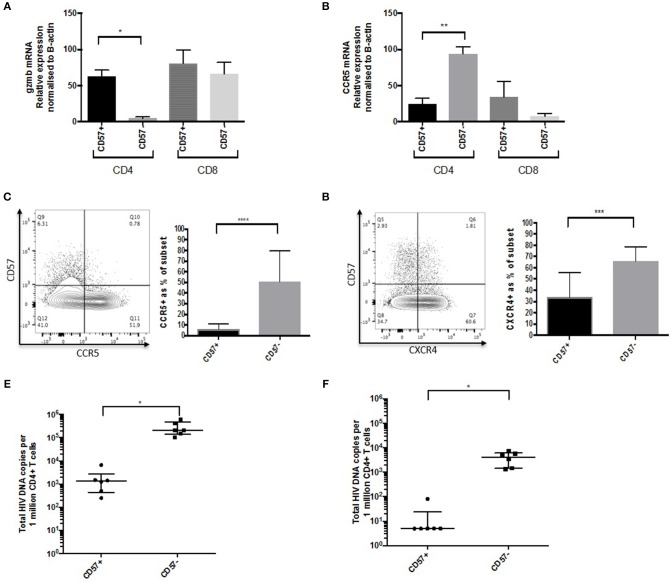
CD57+ CD4 CTLs are not a component of the HIV reservoir. **(A)** *GzmB* mRNA levels in CD57+ and CD57– in CD4 and CD8 subsets. **(B)** *ccr5* mRNA levels in CD57+ and CD57– in CD4 and CD8 subsets. **(C)** CCR5 protein in CD57+ and CD57– in CD4+ in untreated PHI. **(D)** CXCR4 protein in CD57+ and CD57– in CD4+ in untreated PHI (*n* = 12). **(E)** Total HIV DNA untreated PHI. **(F)** Total HIV DNA PHI 1-year post-ART. *p < 0.05, **p < 0.01, ***p < 0.001, ****p < 0.0001.

Frequencies of CD57+ CD4 CTLs increased with chronic HIV infection, which suggests that they may be more resistant to HIV infection and activation-induced apoptosis. We therefore investigated the expression of HIV entry co-receptors CCR5 and CXCR4. mRNA expression levels were analyzed using RT-PCR and *GzmB* was used as a positive control. *GzmB* mRNA levels were higher in the CD57+ subset compared to CD57– (~16-fold, *p* < 0.05), and was comparable to CD8 mRNA levels (Figure 6A). *CCR5* mRNA levels were surprisingly much lower in CD57+ cells compared to CD57– (~3.5-fold, *p* < 0.01; Figure 6B), and this was also evident at the protein level (~12.6% lower, *p* < 0.0001; Figure 6C). Lower expression of CXCR4 was also observed in CD57+ cells (~2.2-fold lower than CD57–, *p* < 0.001; Figure 6D). Total HIV DNA was performed on sorted cells at baseline and 1 year post-ART. CD57+ cells had lower HIV DNA (~2.3 Log, *p* < 0.05) at baseline compared CD57– (Figure 6E). This was more apparent following ART as the majority of CD57+ cells contained <5 copies/million CD4 after therapy (Figure 6F), suggesting that these cells are not a major contributor to the HIV reservoir.

### Diminished Cytokine Secretion, Proliferation and Killing Ability in HIV Infection

**Figure 7:**
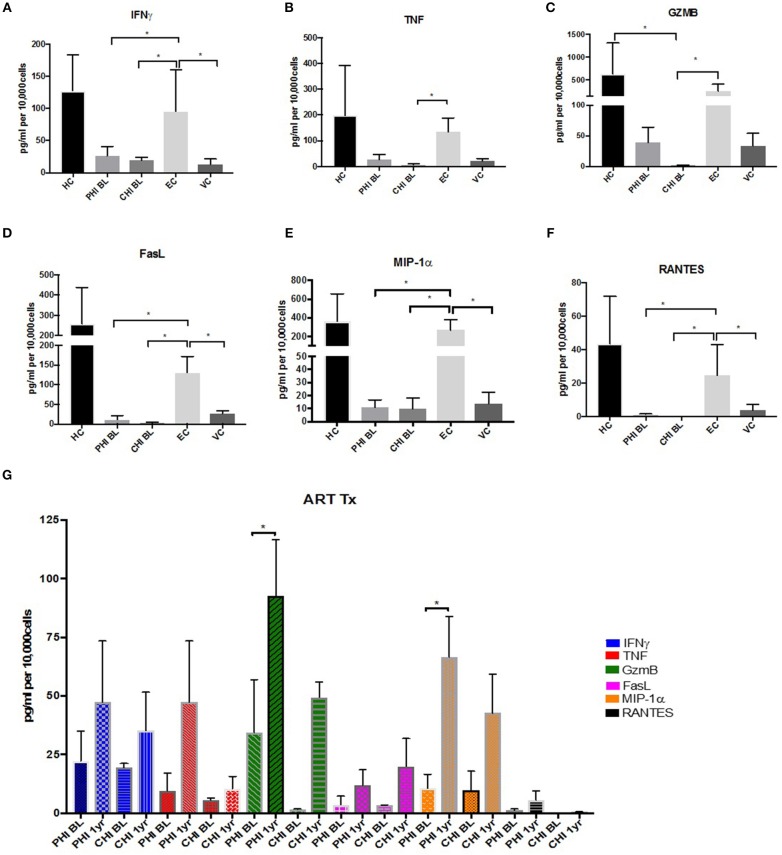
Maintenance of effector cytokine and beta chemokine expression in EC. **(A)** IFNγ **(B)** TNF **(C)** GzmB **(D)** FasL **(E)** MIP1-α **(F)** RANTES expression levels in Healthy controls (HC), PHI and CHI baseline (BL), LTNPs EC and VC. **(G)** Increased expression levels after 1-year ART in PHI and CHI. *p < 0.05.

Cytokine and beta chemokine secretion was examined using Cytokine Bead Array (CBA) assay. CD4+CD57+ T cells from healthy controls produced high levels of effector cytokines IFNγ and TNF, with EC expressing similar levels; whereas CD57+ CTLs from other untreated HIV+ cohorts expressed very low levels of these cytokines (Figures 7A,B). A similar pattern was observed when detecting cytolytic molecules associated with killing, e.g., GzmB and Fas Ligand (FasL) (Figures 7C,D). Beta chemokines MIP-1α (CCL3) and RANTES (CCL5) were highly expressed in CD4+CD57+ cells from HC and EC. This was not the case in PHI, CHI or VC (Figures 7E,F). Significant increase in GzmB (~3.6-fold, *p* < 0.05) and MIP-α (~2.4-fold, *p* < 0.05) secretion levels were observed in PHI post-ART (Figure 7G).

The inability of CD4 CTLs to produce these important molecules may be indicative of overall dysfunction. When bulk PBMCs were stimulated with pooled Gag peptides, no proliferation was observed in the CD4+CD57+ T cells from PHI donors 1-year post ART (Supplementary Figure 7A). This may be due to dysfunction of CD57+ cells early in HIV infection. Killing potential of CD57+ CTLs was examined by sorting CD57+ as effector cells and autologous CD19+ B cells are target cells. Co-culture was activated for 3.5hrs with anti-CD3 beads and caspase-3 expression within target cells was measured by flow cytometry (Supplementary Figure 7B). Background levels of Caspase-3 in target cells alone ranged from 0.5 to 1.21%. CD57+ cells from healthy controls were able to kill ~28% target cells when 3:1 ratio of effector to targets was used (Supplementary Figure 7C). Reduced killing was observed in ART-treated PHI (Supplementary Figure 7D) and CHI (Supplementary Figure 7E) donors compared to healthy controls (1.7-fold less killing for PHI and 17-fold less killing for CHI, when E:T ratio of 3:1 was used).

### A Small Percentage of CD57+ CD4 CTLs From Elite Controllers Are HIV-Specific and Maintain Tbet^high^Eomes+ Phenotype

**Figure 8:**
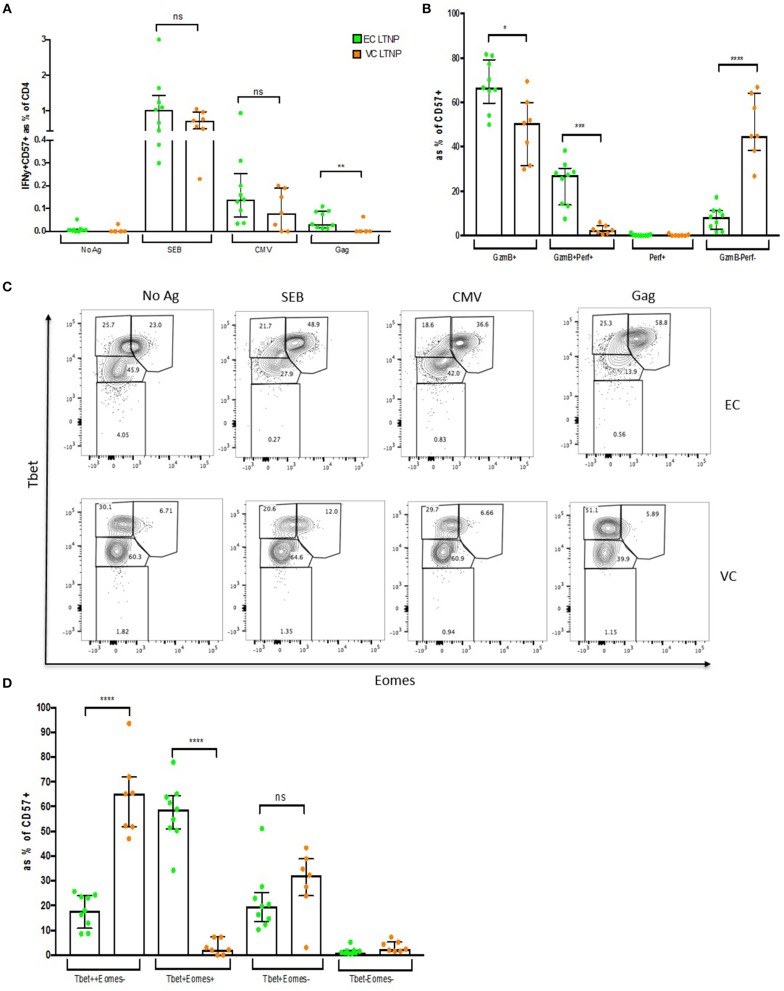
Maintenance of HIV-specific CD57+ CD4 CTLs in EC. **(A)** Higher IFNγ expression within CD57+ cells in ECs compared to VCs. **(B)** High Gzmb+ and GzmB+Perf+ levels in ECs. **(C)** Representative dot plot showing Tbet and Eomes expression between ECs and VCs. **(D)** Maintenance of Tbet ^high^Eomes+ in ECs. *p < 0.05, **p < 0.01, ***p < 0.001, ****p < 0.0001.

To determine HIV specificity of CD57+ CD4 CTLs from LTNP donors, ICS was performed using pooled Gag peptides and CMVpp65 was used as a control antigen. CD57+ cells from EC were able to respond to Gag by IFNγ production (Supplementary Figure 8A). No difference was observed in SEB or CMVpp65 responses between EC and VC, although EC had significantly higher IFNγ production on Gag stimulation (median of 0.031%, *p* < 0.01; Figure 8A). Equivalent IFNγ responses were observed within the CD57– subset (EC = 0.089% and VC = 0.05%; respectively) (Supplementary Figure 8B). An activated cytolytic phenotype based on GzmB+ and GzmB+Perf+ was more evident in EC compared to VC (Figure 8B). Maintenance of IFNγ and cytolytic granule function may be attributed to the increased Tbet^high^(++)Eomes+ phenotype that occurred in EC donors after antigenic stimulation (~28.5-fold, *p* < 0.0001; Figure 8C). Tbet^high^Eomes+ expression in EC was ~11-fold (*p* < 0.0001) higher after Gag stimulation compared to VC (Figure 8D) suggesting that CD57+ CTLs from VCs were unable to up-regulate Tbet and Eomes expression. Collectively, this data suggests that EC were able to maintain functional cytolytic CD4+ T cells compared to other HIV-infected cohorts.

## Discussion

Cytolytic CD4+ T cells are currently under intense investigation regarding their role during viral/bacterial infections, autoimmune conditions and solid tumor malignancies (4). A hindrance to studying this T cell subset can be attributed to the lack of subset specific surface markers that are yet to be identified. Recently, two molecules CRTAM (class-I-restricted T cell-associated molecule) (18) and SLAMF7 (CD319) (19) have been described that identify a subset of CD4+ T cells with cytolytic properties. However, expression of these molecules seems to be transient and tissue specific, and their use as a marker of CD4 CTLs for functional studies is limited. CD57, also called HNK-1, LEU-7, or L2, is an extracellularly expressed terminally sulfated carbohydrate that was first reported as a marker of natural killer cells (20). CD57, as a carbohydrate, has several binding partners involved in immune activation, including neural cell adhesion molecule, ICAM5, NCAM, integrins, L-selectin, and P-selectin (21, 22). Johnson et al. (8) showed that CD4+CD57+ cells co-expressed cytolytic granules and this is supported by our findings. Increased CD57 expression has been associated with CMV seropositivity (3, 9). CD57+ CD4+ cells predominantly expressed granzyme B and to a lesser extent granzyme B together with perforin in healthy donors. This cytolytic phenotype was further heightened in HIV infection with most of the CD57+ subset having activated cytolytic granule expression.

CD57+ CTLs represent a distinct CD4 T cell subset that are mostly effector memory cells that lack CD28 expression, indicative of their cytolytic potential (23, 24). Cytolytic granule expression increased with activation, as did the degranulation marker CD107a. These cells lacked the expression of the IL-2Ra, have low CD127 (IL-7R) expression and do not express CXCR3 *ex vivo*. RNAseq revealed a cytolytic-like transcription profile within CD4 CTLs that are comprised of many cytolytic genes i.e., *gzma, gzmb, prf1, faslg, ccl4, tbx21*, and *eomes*. The transcriptome of CD4 CTLs highly correlated with the CD8 gene signature, together with increased expression of the CD8 lineage transcription factor RUNX3 (25) demonstrate a close relationship between CD4 and CD8 CTLs.

CD57+ cells are dramatically increased in HIV infection—up to 50% of CD4 T cells in some donors. These cells were highly activated with exaggerated cytolytic granule expression *ex vivo*, in both acute and chronic HIV infection. Their increased frequency and persistence during HIV infection may be due to low expression of HIV co-receptors CCR5 and CXCR4. CCR5 is initially required for HIV entry during acute infection and seeding of the latent HIV reservoir (26–28). Low expression of this receptor may make these cells less likely to become infected and less prone to HIV induced apoptosis and thus maintain their frequency in blood. This is also consistent with the relatively low HIV DNA levels found in CD57+ CTLs compared to CD57– that shows that they are not a major part of the HIV reservoir, confirming the previous observation by Brenchley et al. (29).

The progressive decline in functional memory CD4+ T cells has long been a hallmark of chronic HIV infection. Although CD57 CTLs were preserved in HIV infection, their function has been jeopardized. CMV-specific responses via the expression of IFNγ together with antigen-induced proliferation were observed in healthy controls. No proliferation was observed in PHI after 1 year post therapy in response to CMV to HIV Gag. CD57+ cells from HC were able to kill target cells after TCR stimulation. This killing ability was substantially decreased in those with acute HIV infection and was almost entirely diminished in chronic infection even after treatment with ART. Dysfunction of these cells may be at least in part due to prolonged antigenic activation (30–33).

Elite Controllers are defined as long-term non-progressors that have persistent undetectable viral loads and stable intact CD4 counts (34). Many studies have examined both CD4 and CD8 T cell function within this cohort. It is widely acknowledged that ECs have reduced CCR5 expression, lower activation levels and maintain HIV-specific responses compared to other progressor cohorts (34, 35). This was also observed with CD57 CTLs from ECs. They expressed lower levels of CD38/HLA-DR activation markers, and had reduced ICR (PD-1 and TIM-3) expression when compared to other HIV cohorts including PHI and VCs. They were able to secrete effector cytokines (IFNγ and TNF) and cytolytic proteins (GzmB and FasL) upon stimulation, whereas other HIV cohorts were not able to secrete these molecules. Beta chemokine expression was maintained, which may act in an autocrine manner for self-protection against HIV entry as both RANTES and MIP-1α restriction factors can bind to CCR5 (36), which may explain in-part the high frequencies of these cells observed during CHI.

Having higher CD57 CTLs percentages in blood was not indicative of increased HIV-specific CD57 CTLs responses. An IFNγ response to Gag stimulation, albeit small (~0.031%), was detected in ECs and not observed in VCs. This coincided with high gzmb+perf+ expression with activation, suggesting maintenance of cytolytic function. Interestingly, increased Tbet^high^Eomes+ expression was observed in ECs and not VCs after antigenic stimulation. The synergistic interplay between transcription factors Tbet and Eomes has been shown to be important during CD4+ T cell differentiation particularly in driving cytolytic CD4 T cell lineage commitment (8). Tbet is required for effector cytokine expression (37) and Eomes is important for cytolytic granule expression (25). The upregulation of both Tbet and Eomes after activation in CD57+ CTLs from ECs may explain a higher level of HIV control and non-progression compared to VCs. Functional HIV-specific CD57+ CTLs together with CD8 CTLs may contribute to full control of HIV viraemia in ECs. Previous findings that may distinguish the two groups include the HLA types of (HLA-B27 and HLA-B57) that are over-represented in ECs compared to viraemic, polyfunctional T cells and broadly neutralizing antibodies (34, 35). This subset may be an important factor that distinguishes EC from VC. HIV-specific CD4 CTLs may work in concert with CD8 T cells, by way of killing infected target cells presenting class II antigens (12), to maintain viral clearance.

There are limitations on the current study, especially the restricted number of samples from ECs. Since these were long-term stored historical samples and the guidelines for HIV therapy have also changed over time, it is not possible to further explore the longitudinal changes in this group. The HIV-specific IFN-γ responses elicited, although significantly above background, were also small (although consistent with changes transcriptional factor expression which were larger), so a prospective and longitudinal study of this subset exploring the breadth of functionality in relation to clinical outcomes would be valuable.

Overall, CD4 CTLs are an important subset that should be considered when investigating effector T cell responses during primary HIV infection, vaccine trials and treatment interruption studies. Further examination of these cells may shed light on an important effector subset that could contribute to the control of HIV infection and may potentially be of interest in strategies for HIV cure.

## Data Availability

The data has been deposited at the Gene Expression Omnibus repository—accession number is GSE134536.

## Ethics Statement

Participants with Primary HIV (PHI) were recruited as either part of the HEATHER (HIV Reservoir targeting with Early Antiretroviral Therapy) cohort or from the SPARTAC (Short Pulse Antiretroviral Therapy at HIV Seroconversion) trial (EudraCT Number: 2004-000446-20). For inclusion in the HEATHER cohort, participants with identified PHI commenced ART within 3 months of diagnosis, and did not have co-infection with Hepatitis B or C. For our study, cryopreserved PBMCs were used from the closest pre-therapy sample to seroconversion (baseline) and from a sample 9–15 months after commencement of ART (1 year). Only Baseline samples were used from the SPARTAC trial, which was a multi-center, randomized controlled trial of short course ART during PHI, the full design of which is described elsewhere (38). Participants with Chronic HIV (CHI) were recruited in Bloemfontein, located within the Mangaung Metropolitan Municipality in the Free State province of South Africa. Most participants had advanced HIV-1 disease progression (as reflected by a CD4 T cell count < 350 cells/μL). All participants were tested for HIV-1 using a point-of- care HIV-1 rapid test or laboratory-based HIV-1 ELISA. Follow up samples were collected at 6 and 12 months post-ART initiation (39). Long-term non-progressors (LTNP) samples were collected at various sites across New South Wales, Australia; samples were processed and stored at St. Vincent's Centre of Applied Medical Research, Darlinghurst. Eligible subjects were HIV+, asymptomatic and diagnosed at least 8 years previous to enrolment, treatment naïve, and had an absolute CD4+ T cell count ≥ 500 cells/μL. Elite controllers (EC) had undetectable viral load (median < 1.7 Log) whilst viraemic controllers (VC) had detectable viral load (median < 5.8 Log) (Table 1). The St. Vincent's Research Ethics Committee (EC00140) approval number: HREC/12/SVH/298, SVH 12/217. PBMC obtained from healthy donors were approved by the Sheffield Research Ethics Committee (reference 16/YH/0247). All participants from each of the above mentioned cohorts gave informed consent for their participation in these studies.

## Author Contributions

The experiments were conceived and designed by CP, PK, and JF. Experiments were performed by CP and DA. Data analyzed by CP and EM. Design, recruitment of the trial samples were performed by JF, SF, LM, CV, DG, CM, and AK, with trial management performed by JM. The paper was written by CP with input from all authors.

### Conflict of Interest Statement

The authors declare that the research was conducted in the absence of any commercial or financial relationships that could be construed as a potential conflict of interest. The reviewer IO and handling editor declared their shared affiliation.

## Supplementary Material

Supplementary Figure 1 Sort purity. **(A)** gating strategy and post sort purity of CD45O+CD57– cells.

Supplementary Figure 2 Cytolytic granules and chemokine receptor expression in CD57+ cells. **(A)** Gzm A, GzmB and Perf expression in CD57+ and CD57– subsets. **(B)** CD25 and CD127 expression on CD57+ (Blue) within CD3+CD4+ cells (Pink). **(C)** CCR6 and CCR4 expression on CD57+ (Blue) within CD3+CD4+ cells (Pink). **(D)** CXCR3 expression on CD57+ (Blue) within CD3+ CD4+ cells (Pink). ^*^*p* < 0.05.

Supplementary Figure 3 CD57+ CD4 CTLs function. **(A)** CD107a upregulation in CD57+ cells after stimulation. Co-expressed with gzmB and IFNγ. **(B)** IL-2, IL-12 and IL-18 exogenous cytokines required for proliferation of CD57+ CD4. **(C)** IL-2+IL-12 required together with TCR (anti-CD3) stimulation for CD57 proliferation. **(D)** No CD57 upregulation after anti-CD3 stimulation in CD57– subset.

Supplementary Figure 4 Runx3 and ThPOK expression in CD57+ subset. **(A)** *runx3* mRNA expression (Column plot), and RUNX3 protein expressed as MFI **(B)**. **(C)** *Thpok* mRNA expression in CD57+ and CD57–. ^*^*p* < 0.05.

Supplementary Figure 5 CD28 and memory phenotype of CD4 CTLs. **(A)** Reduced CD28 expression with HIV infection. **(B)** Representative dot plot showing memory subset transitions within CD57+ subset during chronic HIV infection; Naïve (CCR7+CD45RA+), Central memory Tcm (CCR7+CD45RA–), Effector memory Tem (CCR7–CD45RA–) and terminally differentiated Temra (CCR7– CD45RA+). **(C)** Tcm to Tem transition of CD57+ cells in HIV infection. ^*^*p* < 0.05, ^***^*p* < 0.001.

Supplementary Figure 6 CD57+ CD4 CTLs phenotype. **(A)** CD57 vs. CD38, HLA-DR, PD-1 and TIM-3 expression dot plots. **(B)** No changes in Activation or Inhibitory markers 1-year post-ART in PHI. **(C)** No difference in Tbet and Eomes expression after ART treatment. **(D)** Representative dot plots showing CD57 vs. CX3CR1, SLAMF7, 2B4 (CD244) and CrTAM expression in CD3+CD4+. **(E)** CTLs extracellular marker expression. ^*^*p* < 0.05.

Supplementary Figure 7 CD57+ dysfunction in HIV infection. **(A)** Dysregulation of CD57+ proliferation in ART treated primary HIV donors. **(B)** Caspase-3 upregulation in Target cells (T) after anti-CD3 activation of CD57+ Effector cells (E). **(C–E)** Killing of target cells by CD57+ Effectors in Healthy controls, ART treated PHI and ART treated CHI, respectively. ^*^*p* < 0.05, ^**^*p* < 0.01, ^****^*p* < 0.0001.

Supplementary Figure 8 Maintenance of CD57+ function in EC. **(A)** Dot plot representing IFNγ expression 6 h post stimulation with SEB, CMV and gag. **(B)** No difference in IFNγ expression between EC and VC in CD57– subset.
