# Supplementary figures and images for: Maintenance of Functional CD57+ Cytolytic CD4+ T Cells in HIV+ Elite Controllers
Source: Front Immunol. 2019 Aug 8;10:1844. doi: 10.3389/fimmu.2019.01844 (PMC6694780; doi:10.3389/fimmu.2019.01844)

# Suppl. Fig.1

A)

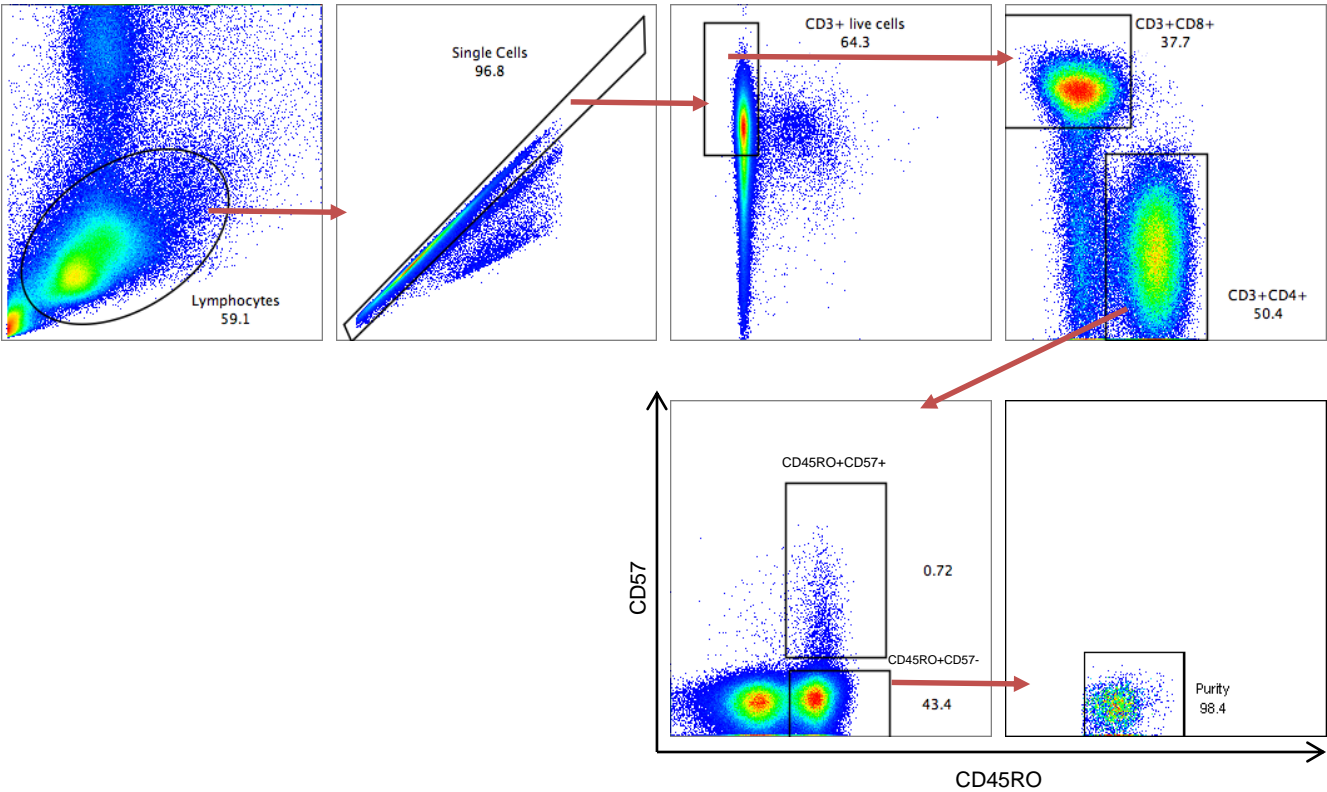

Suppl. Fig.2

A)

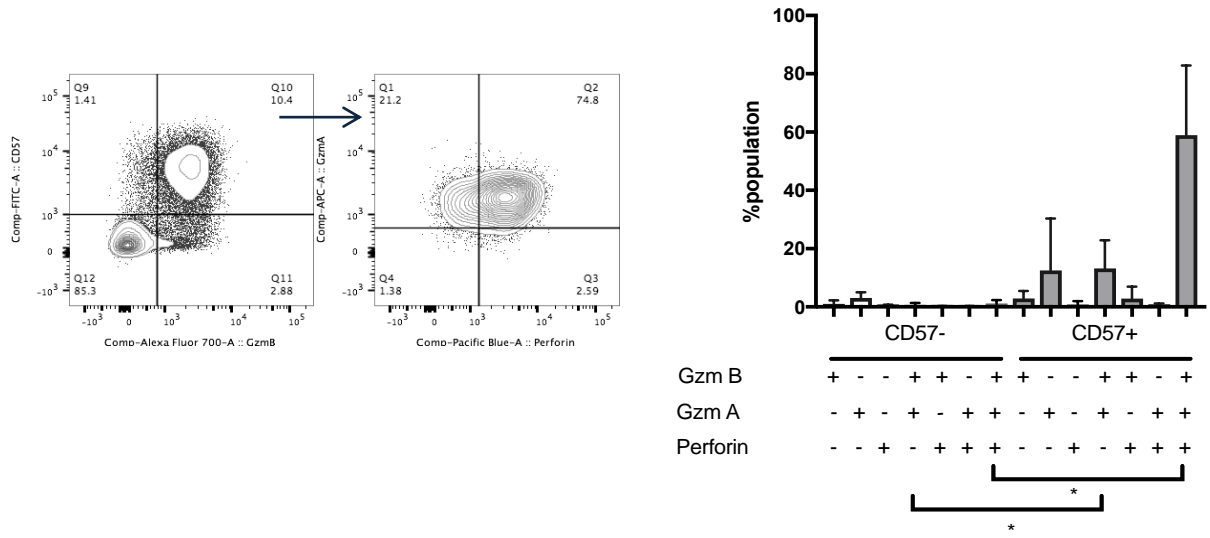

B)

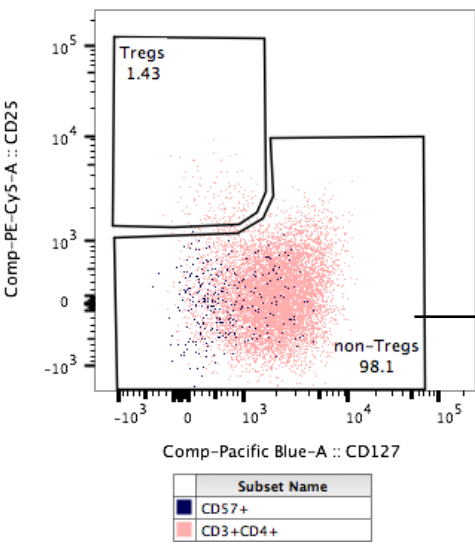

C)

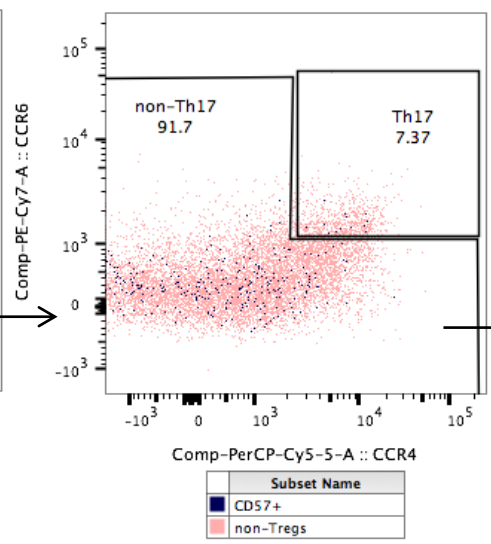

D)

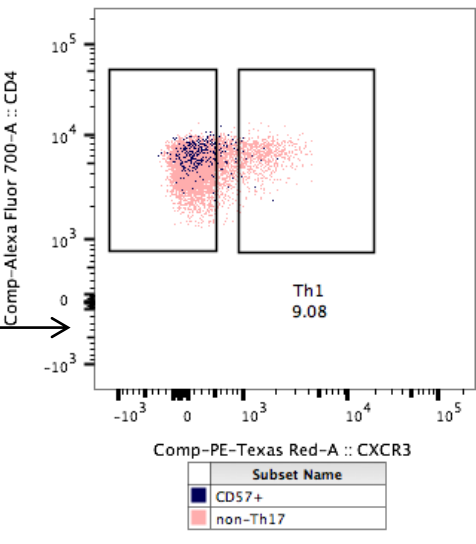

# Suppl. Fig.3

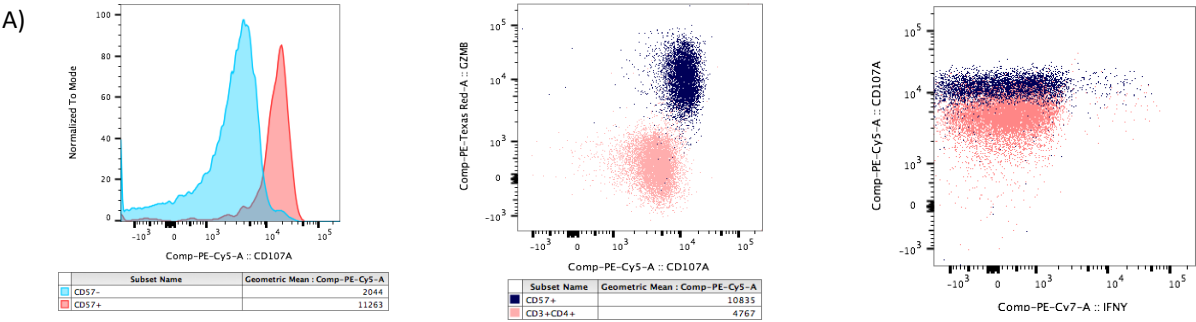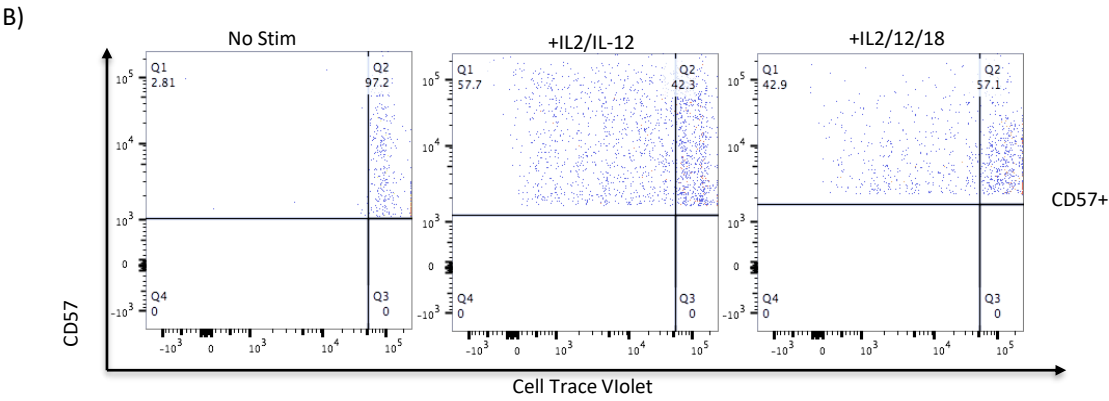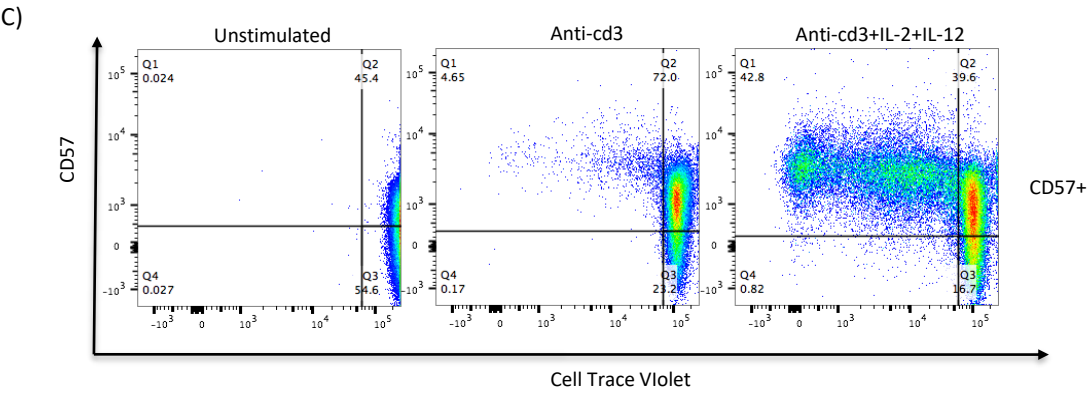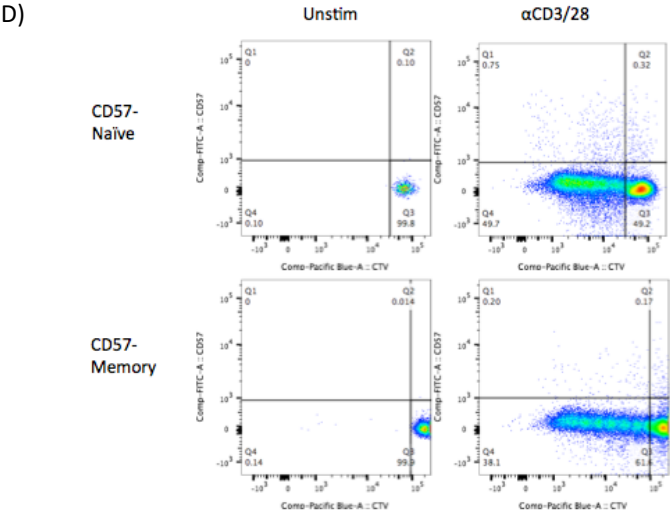

# Suppl. Fig.4

A)

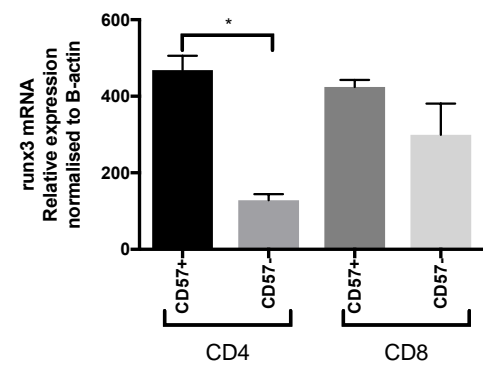

B)

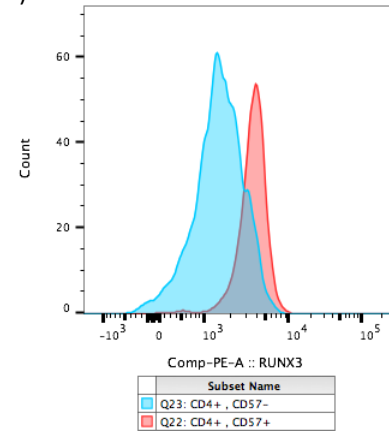

C)

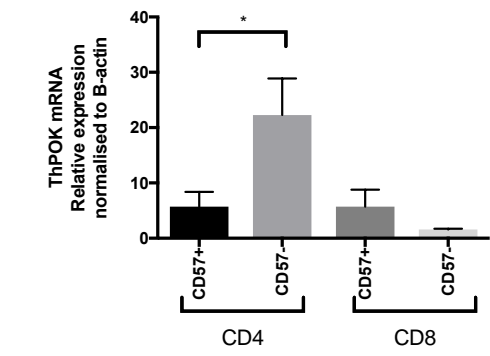

# Suppl. Fig.5

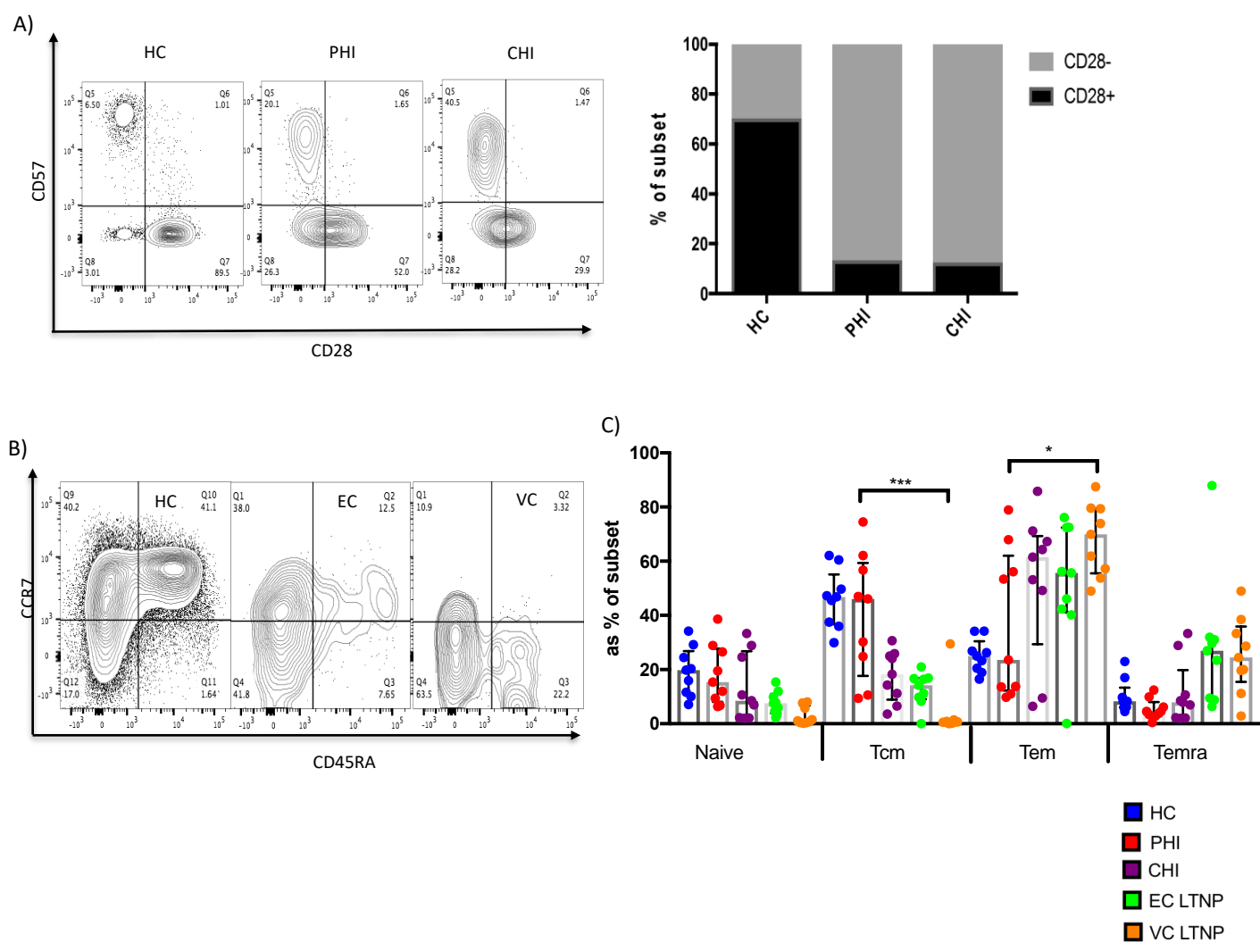

Suppl. Fig.6

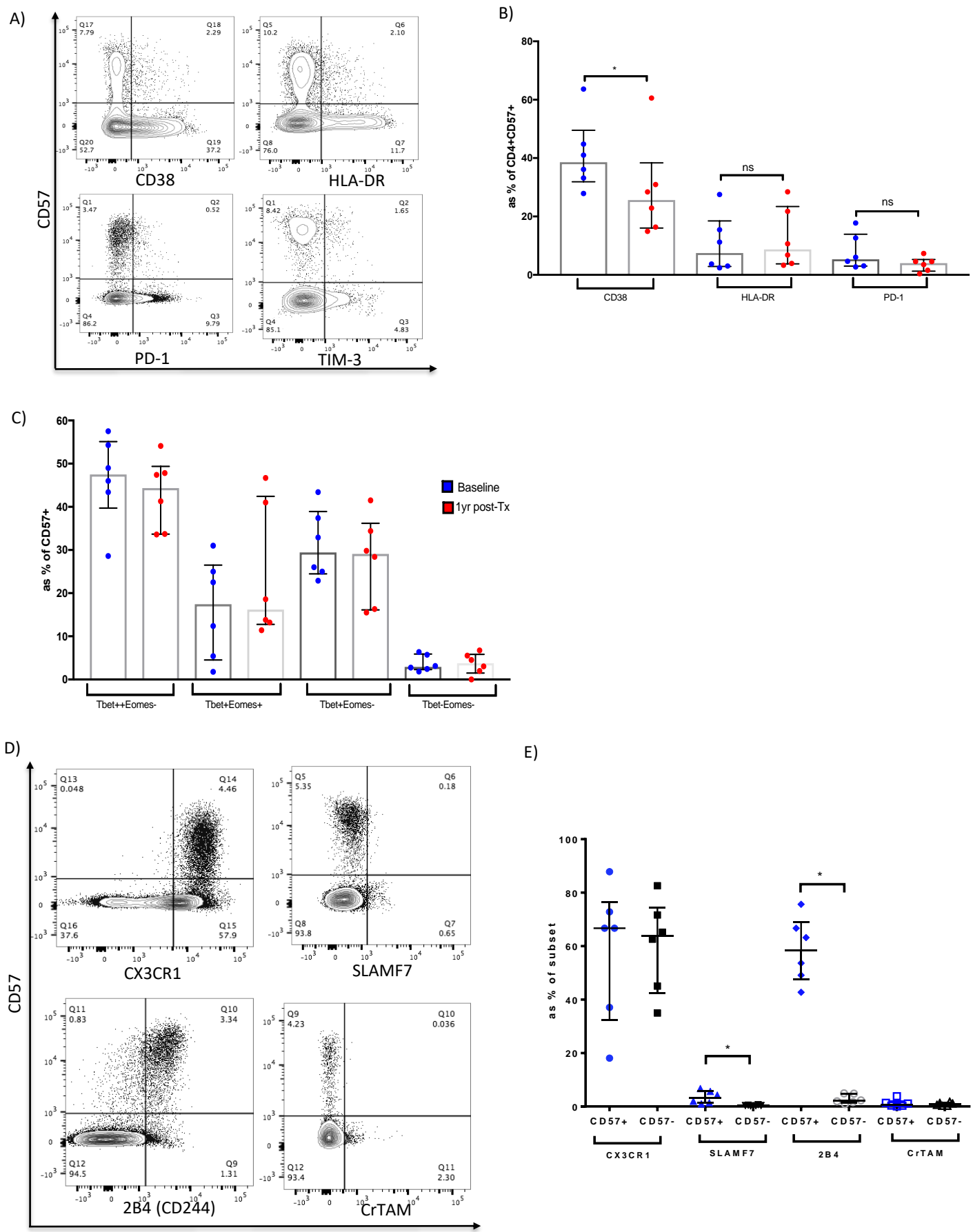

# Suppl. Fig.7

A)

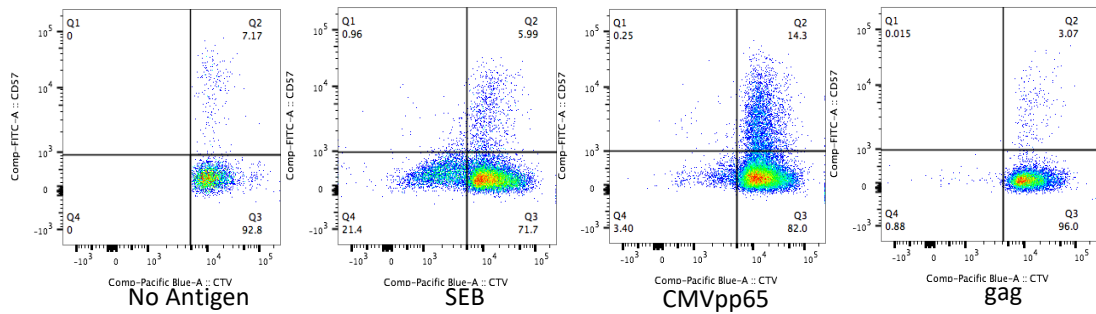

B)

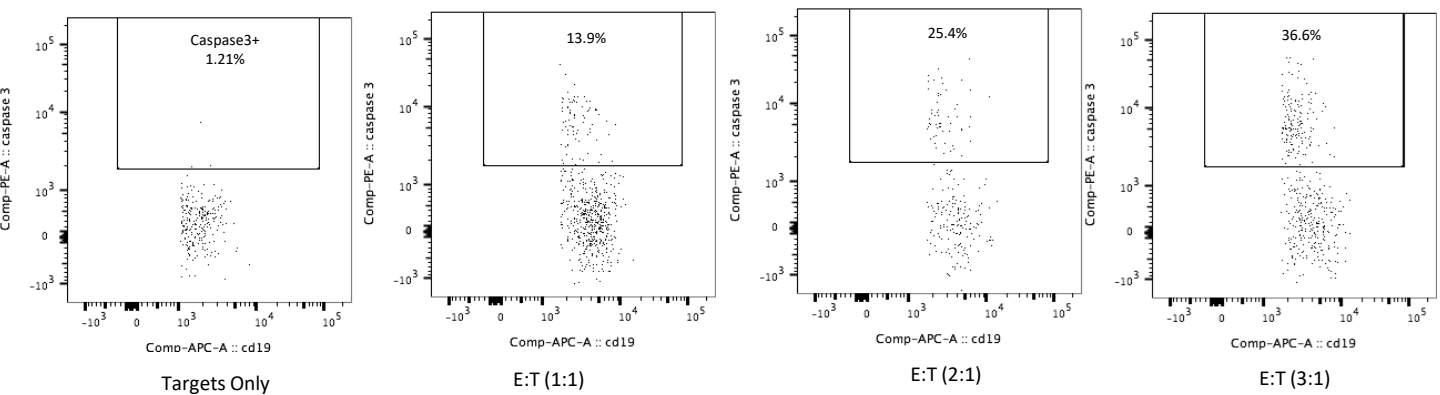

C)

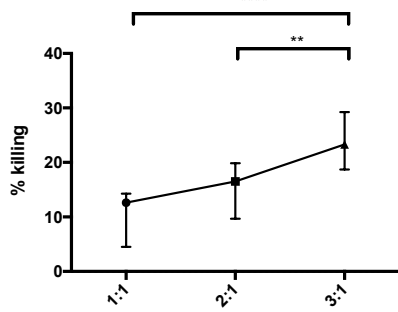

D)

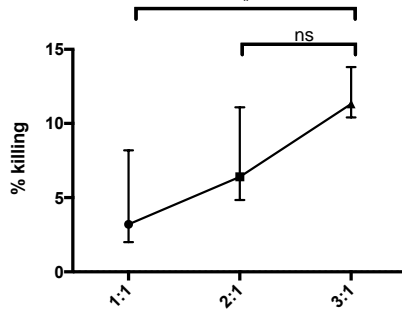

E)

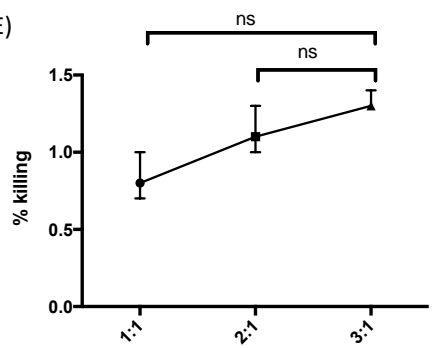

# Suppl. Fig.8

A)

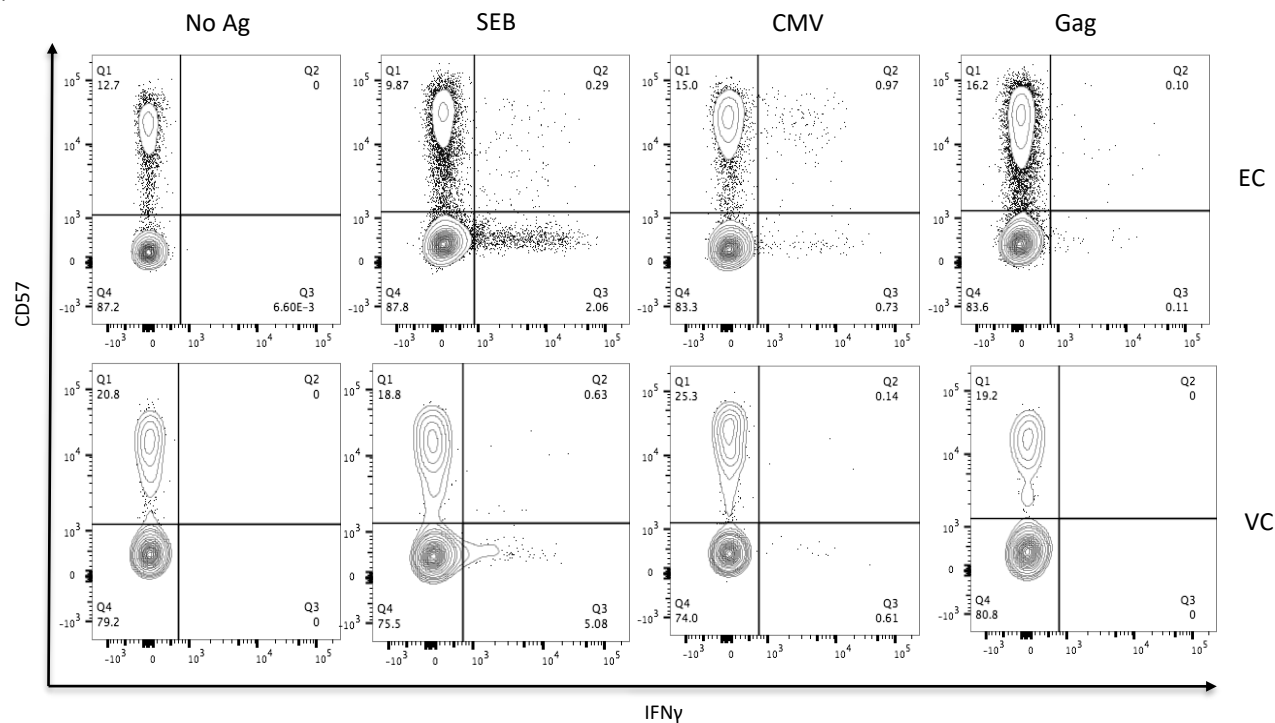

B)

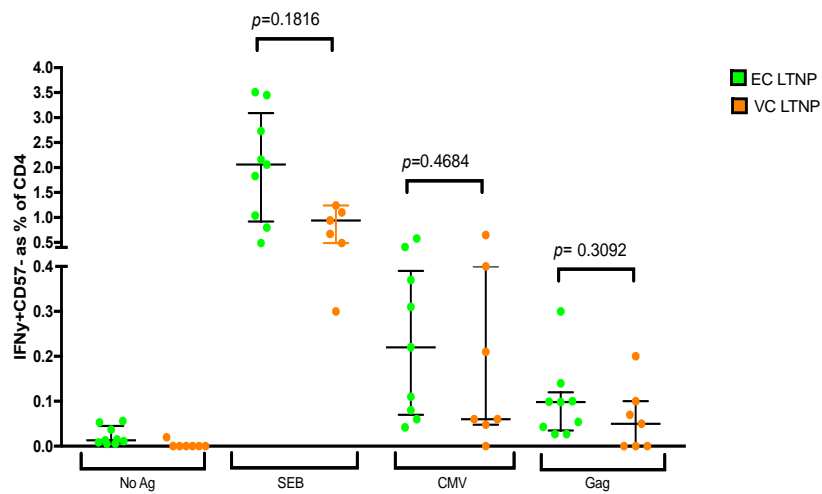

Supplement: Supplementary Figure 1 — Sort purity. (A) gating strategy and post sort purity of CD45O+CD57– cells. [file Data_Sheet_1.pdf]
